# Prognostic Value of Preoperative Hydronephrosis in Patients Undergoing Radical Nephroureterectomy for Upper Tract Urinary Carcinoma: A Systematic Review and Meta-Analysis

**DOI:** 10.3389/fonc.2020.600511

**Published:** 2020-12-11

**Authors:** Tao Ye, Xiaoqi Yang, Peng Lv, Haoran Liu, Zhangqun Ye

**Affiliations:** Department of Urology, Tongji Hospital, Tongji Medical College, Huazhong University of Science and Technology, Wuhan, China

**Keywords:** preoperative hydronephrosis, prognostic value, upper tract urinary carcinoma, radical nephroureterectomy, meta-analysis

## Abstract

**Background:**

Several recent publications have evaluated the prognostic value of preoperative hydronephrosis (HN) in patients with upper tract urinary carcinoma (UTUC). The aim of this meta-analysis was to explore the pooled effect of preoperative HN on the prognosis of UTUC patients treated with radical nephroureterectomy (RNU) based on current evidence.

**Methods:**

We performed a systematic search of Pubmed, Cochrane library, and Web of Science databases from inception to June 2020. The outcomes of interest included overall survival (OS), cancer-special survival (CSS), disease-free survival (DFS), and intravesical recurrence-free survival (IVRFS).

**Results:**

Twenty-two studies with a total of 7,542 patients satisfied the eligibility criteria and were finally included in this meta-analysis. The percent of patients with preoperative HN varied in the eligible studies, ranging from 18 to 81%. The pooled results showed that preoperative HN was significantly associated with worse OS (*P* = 0.004), CSS (*P* < 0.001), and DFS (*P* = 0.005), but not IVRFS (*P* = 0.12). No obvious publication bias was detected by Begg’s test in all the analyses.

**Conclusions:**

The results drawn in our meta-analysis suggest that the presence of preoperative HN is associated with worse prognosis in patients treated with RNU for UTUC. Therefore, closer surveillance and more aggressive therapy may be needed for UTUC patients present with preoperative HN. Well-designed prospective studies are necessary to substantiate the prognostic value of HN in UTUC.

## Introduction

Upper tract urothelial carcinoma (UTUC) is a relatively uncommon malignancy, accounting for only 5 to 10% of all urothelial carcinomas (1, 2). UTUC can arise anywhere along the urinary tract epithelium from renal calyces to ureteral orifice; most of the tumors are located in the renal pelvis, and only 30% occur in the ureter (3). In spite of advances in minimally invasive treatments, radical nephroureterectomy (RNU) with bladder cuff removal still remains the ‘gold-standard’ treatment for UTUC, mainly *via* either an open or a laparoscopic approach (1). However, the prognosis of UTUC is generally not good after radical surgery due to the high possibility of invasive diseases at diagnosis.

Currently, many studies have discussed the prognostic factors for UTUC undergoing RNU, and accumulating knowledge of the factors would help the urologists better evaluate the outcome of UTUC, for a more effective therapy overall. Neutrophil-to-lymphocyte ratio (NLR), a blood-based biomarker, was reported to correlate with worse outcomes in various malignant tumors; a previous meta-analysis summarized that high preoperative blood-based NLR was obviously associated with poorer OS, RFS, and CSS in UTUC patients who underwent RNU (4). Ku et al. also reported that lymphovascular invasion (LVI) could be used as a potential predictor of mortality in UTUC (5). In addition, tumor stage, tumor grade, and lymph node (LN) status have been established as the major prognostic factors for UTUC, demonstrating the heterogeneity and aggressiveness of this type of cancer (6–8).

Preoperative hydronephrosis (HN) status can be detected in patients with bladder cancer or UTUC. For bladder cancer patients, a meta-analysis summarized that the presence of preoperative HN was significantly correlated with worse OS and CSS after radical cystectomy (9). Recently, several studies also have indicated that preoperative HN may be a potential prognostic predictor for UTUC after RNU, but its effect has not been fully understood and their results are still in controversies (10, 11). As it is easily available for the detection of preoperative HN in clinical practice, a better understanding of HN may improve the oncological outcomes for UTUC patients after RNU. Thus, we performed a literature review and meta-analysis to further explore the generalized impact of preoperative HN on the prognosis of patients who underwent RNU for UTUC.

## Materials and Methods

### Search Strategy

This work was reported in line with PRISMA (Preferred Reporting Items for Systematic Review and Meta-analyses) (12). Published studies were identified from Pubmed, Cochrane library, and Web of Science databases (last search date: June 2020), and additional articles were found by screening the reference lists of the retrieved records. Only original studies written in English and published after January 2010 were included. The following terms were combined to perform the electronic search: ‘upper tract urothelial cancer’ or ‘transitional cell carcinoma of the upper urinary tract’, ‘radical nephroureterectomy’, ‘prognosis’ or ‘survival’ or ‘oncological outcome’, and ‘hydronephrosis’. Two independent investigators (TY and XQY) screened the titles and abstracts of retrieved articles, and disagreements were settled by negotiation. The protocol was registered in the International Prospective Register of Systematic Reviews database (PROSPERO: CRD42019132011).

### Inclusion and Exclusion Criteria

Studies satisfying the following criteria were included: (i) studies that evaluated the association between preoperative HN and oncological outcomes in patients who underwent RNU for UTUC; (ii) studies that directly reported hazard ratios (HRs) with their corresponding 95% CIs of overall survival (OS), cancer-special survival (CSS), disease-free survival (RFS), and intravesical recurrence-free survival (IVRFS) in multivariable logistic regression analysis; (iii) studies that enrolled more than 100 participants, and (iv) the median follow-up >12 months. The exclusion criteria were as follows: (i) the literatures were non-original articles, comments, reviews, or meta-analysis; (ii) studies that included patients with recurrent UTUC, metastatic carcinoma, or receiving neoadjuvant chemotherapy were excluded; and (iii) (potentially) overlapping study populations were reported for the same outcome.

### Data Extraction and Quality Assessment

A standardized-items form was used by two independent reviewers (TY and HL) to extract usable data from eligible full length articles. In our meta-analysis, OS and CSS were defined as the interval between surgery and any cause death and cancer-caused death, respectively; DFS was defined as the interval between surgery and local relapse or distant metastasis, excluding the recurrence in bladder, and these studies in which DFS was defined as recurrence both in bladder and non-bladder lesions were not considered for DFS analysis; IVRFS was defined as the interval between surgery and the recurrence in the bladder. The following information was recorded: first author’s name, publication year, country/region of origin, study period, study design, sample size, the number of patients with HN, patient characteristics (age and gender), follow-up duration, outcomes (OS, CSS, DFS, and IVRFS), tumor characteristics (location, size, pT stage, pN stage, and grade), and treatment management. Newcastle-Ottawa Scale was applied to determine the quality of the included studies (13). The score ≥6 was considered high-quality. Disagreements were discussed to reach a consensus.

### Statistical Analysis

Stata 12.0 statistical software (Stata Corp., College Station, TX) was applied to perform all the data analyses. HRs with their 95% CIs of OS, CSS, DFS, and IVRFS in multivariable logistic regression analysis from each study were used to obtain the combined HRs. A random-effect or fixed-effect model was chosen to pool the results based on the between-study heterogeneity. *I^2^* statistics >50% and chi-squared test *P* value <0.1 demonstrated notable heterogeneity. A Galbraith plot was performed and a leave-one-out analysis was conducted to identify studies causing heterogeneity and evaluate their influence on the combined HRs (14). Sensitivity analysis was conducted to examine the stability of the final results and Begg’s test to determine the risk of publication bias among the included studies. P-value lower than 0.05 was considered statistically significant.

## Results

### Summary of the Enrolled Studies

**Figure 1:**
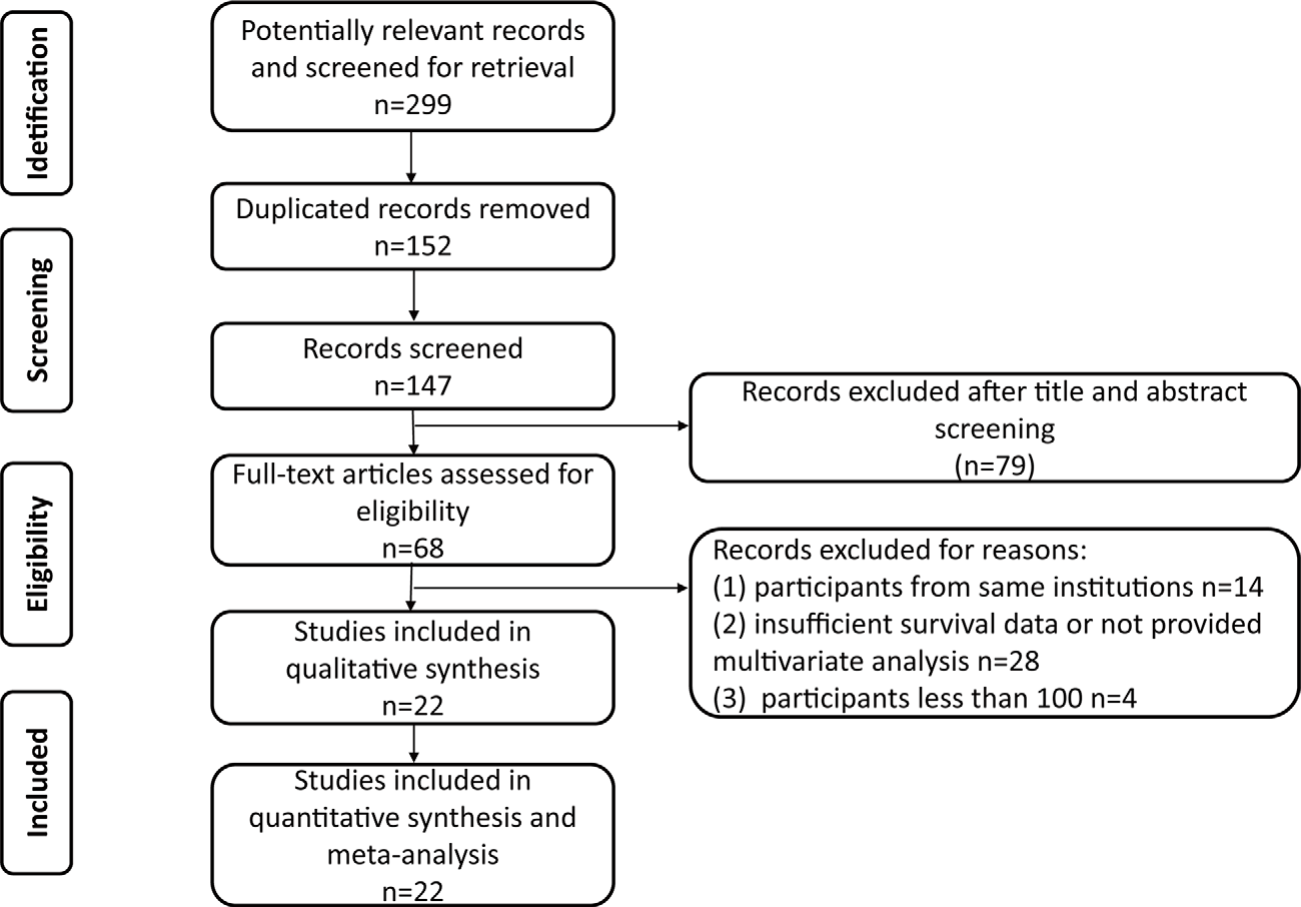
Flow diagram of the study selection process.

The PRISMA flow chart of the search strategy for eligible studies is summarized in **Figure 1**. We assembled a total of 299 potentially relevant records from the electronic databases, and finally 68 articles were retrieved for full texts. After final evaluation, only 22 studies were deemed fully eligible for this meta-analysis (10, 11, 15–34). The main characteristics and findings of the 22 eligible studies are summarized in **Tables 1**, **2**. Among the 7,542 patients (4,868 males and 2,674 females), the status of HN was confirmed in 3,867 of them, and the percent of patients with preoperative HN in each study ranged from 18 to 81%. As for the HN evaluation method, eight studies reported directly, while the others were not in the published papers. All the included studies were retrospective design, with a wide recruitment period from 1990 to 2018. The enrolled UTUC patients were from different countries or regions (China, Japan, USA, Korea, Taiwan, Canada, Germany, and France) with the median follow-up duration ranging from 24.8 to 67.8 months. The quality assessment of eligible studies by the Newcastle-Ottawa scale showed that all the included studies were high-quality with the scores ≥6 (**Table 1**).

**Table 1:** Summary of the included studies in this meta-analysis (individual study characteristics).

Studies	Country/Region	Study period	Study design	Patients, n	HN, n (%)	HN evaluation	Age, yr, median (range)	Follow-up, mo, median (range)	Outcomes	NOS
Chen et al. (15)	China	2008–2018	RTP, SC	232	142 (61)	NR	65 (IQR: 58–73)	39 (IQR: 17–53)	OS, CSS, DFS	8
Huang et al. (16)	China	2000–2016	RTP, SC	133	92 (69)	NR	66 (36–86)	NA	OS, CSS, DFS	7
Itamin et al. (17)	Japan	1995–2016	RTP, MI	125	64 (51)	NR	72 (38–90)	51 (IQR: 6–227)	CSS	7
Freifeld et al. (18)	USA	1993–2016	RTP, MI	245	71 (31)	NR	70 ± 9.8^a^	27	DFS	6
Son et al. (19)	Korea	2004–2015	RTP, MI	1,137	524 (46)	NR	69 (IQR: 61–74)	39 (IQR: 18–64)	CSS	7
Tan et al. (20)	China	2003–2015	RTP, SC	620	376 (61)	NR	65.7 ± 11.4^a^	51 (1–168)	OS, CSS, DFS	8
Kohada et al. (21)	Japan	1999–2016	RTP, SC	148	68 (46)	CT	71 (IQR: 64–78)	35.5 (IQR: 12–66)	CSS, DFS	8
Fang et al. (22)	China	1999–2011	RTP, SC	612	339 (55)	CT, MRI, US	68 (IQR: 60–74)	64	CSS	6
Jan et al. (23)	Taiwan	2007–2017	RTP, SC	424	344 (81)	NR	70 (IQR: 62–77)	35 (IQR: 14–60)	OS, CSS, DFS	8
Kim et al. (24)	Korea	1991–2012	RTP, SC	452	197 (44)	NR	64.0 ± 10.2^a^	67.8 (0–254)	OS, CSS	7
Liu et al. (25)	China	2000–2013	RTP, MI	265	176 (66)	NR	62.0 ± 10.7^a^	60	OS, CSS, IVRFS	8
Nakagawa et al. (26)	Japan	1996–2013	RTP, MI	109	54 (53)	CT, US	71 (IQR: 64–77)	46.5 (IQR: 23.2 –76.7)	CSS, DFS	8
Lee et al. (27)	Korea	2000–2015	RTP, MI	760	232 (31)	CT, MRI	NA	45 (IQR: 3–76)	IVRFS	7
Liang et al. (28)	China	2001–2014	RTP, SC	172	109 (63)	CT, MRI, US	70 (IQR: 63–77)	44 (IQR: 24–62)	OS, CSS	8
Song et al. (29)	China	2005–2011	RTP, SC	140	82 (59)	NR	67 (39–81)	45 (11–108)	DFS	6
Fradet et al. (30)	Canada	1990–2010	RTP, MI	742	518 (78)	NR	69.7 ± 10.8^a^	24.8 (IQR: 7.7–56.8)	IVRFS	7
Lee et al. (31)	Korea	2001–2010	RTP, SC	138	100 (72)	NR	NA	NA	IVRFS	6
Zou et al. (32)	China	1999–2013	RTP, SC	122	73 (60)	NR	64 (35–80)	53 (3–159)	CSS, IVRFS	7
Aziz et al. (33)	Germany	1992–2012	RTP, MI	242	83 (34)	NR	69 (IQR: 64–76)	30 (IQR: 11–60)	OS, CSS, DFS	8
Zhang et al. (34)	China	2000–2010	RTP, SC	217	110 (51)	CT, MRI, IVU, US	69 (62–81)	52 (IQR: 12–78)	CSS	7
Bozzini et al. (10)	France	1995–2010	RTP, MI	401	74 (18)	CT, MRI, IVU	69 (IQR: 60–76)	26 (IQR: 9–49)	OS, CSS, DFS	8
Ng et al. (11)	USA	1993–2005	RTP, MI	106	39 (37)	CT	NA	47 (1–164)	CSS, DFS	7

HN, hydronephrosis; NOS, Newcastle-Ottawa scale; RTP, retrospective; MI, multi-institutional; SC, single center; IQR: interquartile range; NA, not available; OS, overall survival; CSS, cancer-specific survival; DFS, disease-free survival; IVRFS, intravesical recurrence-free survival; ^a^mean ± SD.

**Table 2:** Summary of included studies (patient, tumor, and treatment).

Studies	Male/Female, n	Tumor location, n	pT Stage, n	pN Stage, n	Tumor grade, n	Surgical approach, n	Adjuvant chemotherapy, n (%)
Chen et al. (15)	132/100	Renal pelvis/Ureter/Both, 87/113/32	T1/T2/T3/T4, 52/70/86/24	N0/N+, 194/38	Low/High, 53/179	ORNU/LRNU, 89/143	NA
Huang et al. (16)	83/50	Renal pelvis/Ureter/Both, 69/50/14	Ta-T1/T2/T3/T4, 50/55/16/12	N0-Nx/N+, 118/15	Low/High, 49/84	RNU	NA
Itamin et al. (17)	96/29	Renal pelvis/Ureter, 68/57	Ta-T1/T2/T3/T4, 54/17/48/6	N0/N+, 117/8	Low/High, 26/99	RNU	37 (29)
Freifeld et al. (18)	152/93	Renal pelvis/Ureter/Both, 116/85/19	NA	NA	NA	RNU	NA
Son et al. (19)	825/312	Renal pelvis/Ureter/Both, 523/422/92	Ta-Tis/T1/T2/T3/T4, 113/332/224/436/32	N0/Nx/N+, 348/757/32	Low/High, 336/801	ORNU/LRNU, 393/744	348 (31)
Tan et al. (20)	355/265	Renal pelvis/Ureter/Both, 350/161/109	Ta-T1/T2/T3/T4, 187/123/218/92	N0/Nx/N+, 82/472/66	Low/High, 158/462	ORNU/LRNU, 442/178	255 (41)
Kohada et al. (21)	112/36	Renal pelvis/Ureter, 82/66	Ta-T2/T3-T4, 82/66	N0/Nx/N+, 29/111/8	G1-G2/G3, 60/88	RNU	25 (17)
Fang et al. (22)	340/272	Renal pelvis/Ureter, 341/271	Ta-T1/T2-T4, 206/406	N0-Nx/N+, 571/41	G1/G2/G3, 19/334/259	RNU	NA
Jan et al. (23)	189/235	Renal pelvis/Ureter/Both, 191/138/95	Ta-T1/T2/T3-T4, 161/83/180	N0-Nx/N+, 399/25	Low/High, 22/402	RNU	40 (9)
Kim et al. (24)	347/105	Renal pelvis/Ureter/Both, 223/165/64	Ta-T1/T2/T3-T4, 187/75/188	N0/Nx/N+, 68/365/19	Low/High, 143/309	RNU	110 (24)
Liu et al. (25)	198/67	Renal pelvis/Ureter/Both, 119/129/17	T1/T2/T3-T4, 85/56/124	N0/Nx, 109/156	Low/High, 103/162	ORNU/LRNU, 213/52	57 (22)
Nakagawa et al. (26)	67/42	Renal pelvis/Ureter/Both, 50/23/36	T3/T4, 104/5	N0/Nx, 21/88	G2/G3, 40/69	RNU	43 (39)
Lee et al. (27)	561/199	Renal pelvis/Ureter/Both, 388/290/82	Ta/T1/T2/T3/T4, 64/264/127/296/9	N0/Nx/N+, 26/711/23	Low/High, 229/531	ORNU/LRNU, 360/400	210 (28)
Liang et al. (28)	105/67	NA	Ta-T1/T2/T3/T4, 39/55/68/10	N0/N+, 156/16	Low/High, 78/94	ORNU/LRNU, 143/29	32 (19)
Song et al. (29)	86/54	NA	Ta-T2/T3-T4, 88/52	NA	G1-G2/G3, 67/73	RNU, 140	0
Fradet et al. (30)	438/304	Renal pelvis/Ureter/Both, 420/161/141	Ta-T1/T2/T3/T4, 331/105/182/45	N0/Nx/N+, 114/571/57	G1/G2-G3, 220/503	ORNU/LRNU, 267/345	73 (10)
Lee et al. (31)	96/42	Renal pelvis/Ureter, 58/80	Ta/T1/T2/T3/T4, 9/41/22/64/2	N0/Nx/N+, 29/99/10	Low/High, 46/92	ORNU/LRNU, 46/102	43 (28)
Zou et al. (32)	87/35	Renal pelvis/Ureter/Both, 72/43/7	T1/T2/T3/T4, 48/48/21/5	NA	Low/High, 66/56	ORNU/LRNU, 101/21	NA
Aziz et al. (33)	153/89	Renal pelvis/Ureter/Both, 133/67/42	Ta-Tis/T1/T2/T3/T4, 60/35/52/91/4	N0/Nx/N+, 80/103/59	G1/G2/G3, 43/57/142	ORNU/LRNU, 226/16	41 (17)
Zhang et al. (34)	130/87	Renal pelvis/Ureter, 146/71	Ta-Tis/T1/T2/T3/T4, 33/50/28/89/17	N0-Nx/N+, 198/19	G1/G2/G3, 23/56/138	ORNU/LRNU, 113/104	NA
Bozzini et al. (10)	249/152	Renal pelvis/Ureter/Both, 264/110/27	Ta-Tis/T1/T2/T3/T4, 121/94/37/122/27	N0/Nx/N+, 116/254/31	G1/G2/G3, 30/160/211	RNU	NA
Ng et al. (11)	67/39	Renal pelvis/Ureter, 69/37	Ta-Tis/T1/T2/T3/T4, 52/19/14/17/4	N0/Nx/N+, 18/85/3	Low/High, 59/47	RNU	0

IQR, interquartile range; NA, not available; RNU, radical nephroureterectomy; ORNU, open radical nephroureterectomy; LRNU, radical nephroureterectomy.

### Meta-Analysis of OS

**Figure 2:**
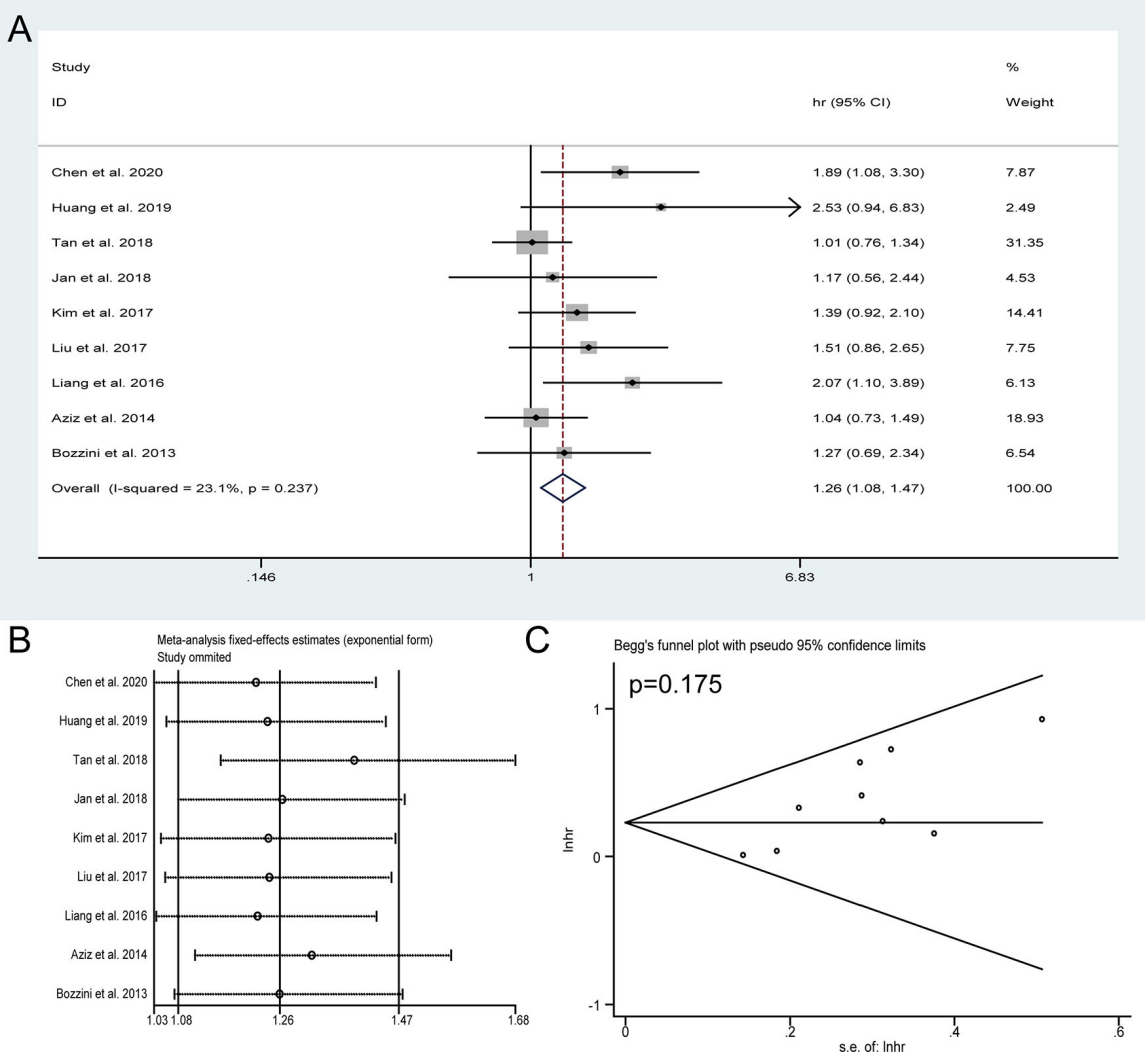
Forest plots of studies to evaluate the relation between preoperative hydronephrosis and overall survival **(A)**. Sensitivity analysis for the meta-analysis of overall survival **(B)**. Begg’s test for the meta-analysis of overall survival **(C)**.

Nine studies with 2,941 patients were performed to explore the impact of HN on the OS of patients with UTUC receiving RNU. Due to no significant heterogeneity observed (I^2^ = 23.1%), a fixed-effect model was used. The results showed that the patients with preoperative HN were subjected to unfavorable OS (HR = 1.26, 95% CI: 1.08 to 1.47, *P* = 0.004) (**Figure 2A**). Sensitivity analysis was conducted to evaluate the outcome stability, and the result verified the robustness of the meta-analysis of OS (**Figure 2B**). No obvious publication bias was detected by Begg’s test (*P* = 0.175) (**Figure 2C**).

### Meta-Analysis of CSS

**Figure 3:**
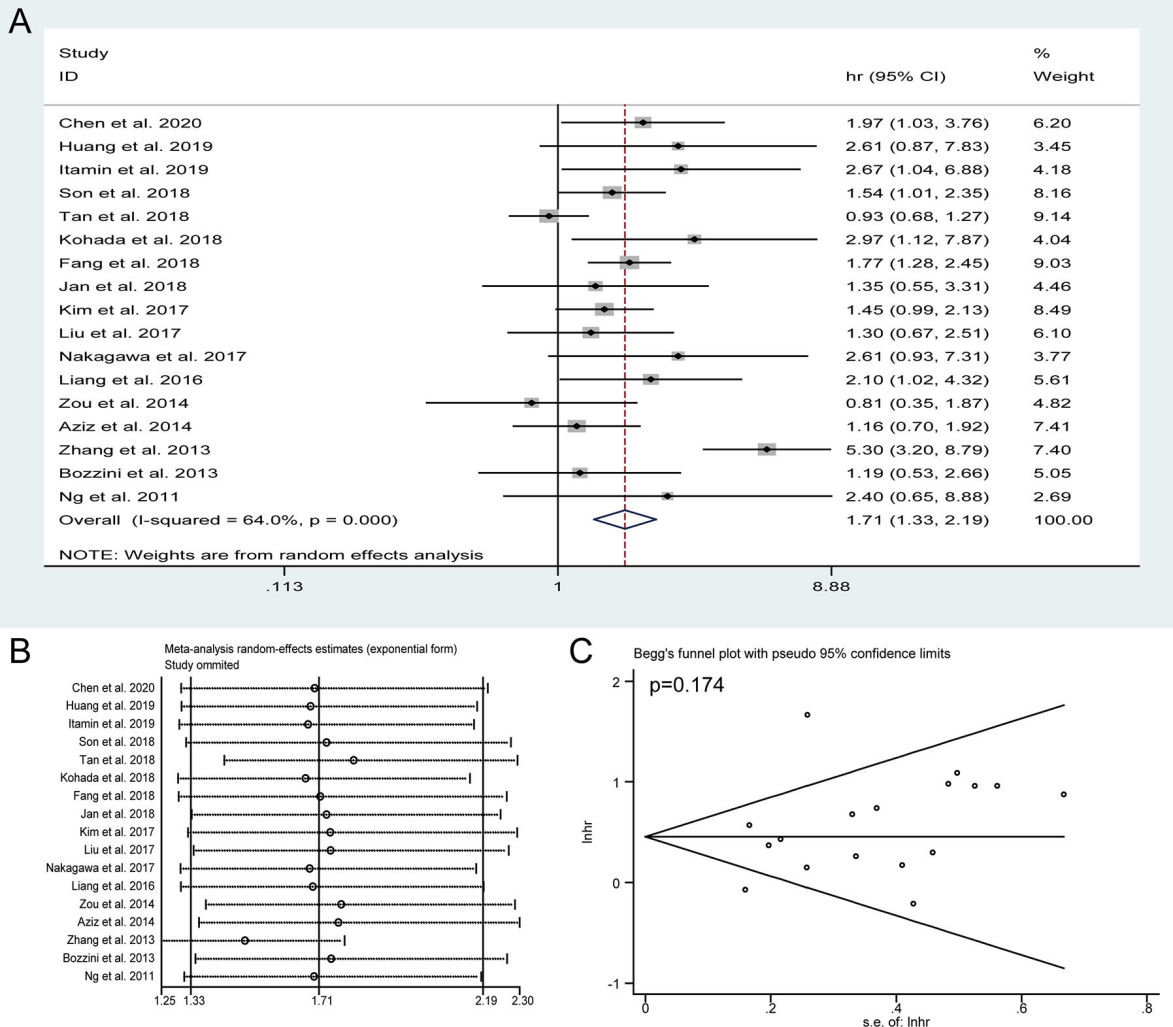
Forest plots of studies to evaluate the relation between preoperative hydronephrosis and cancer-specific survival **(A)**. Sensitivity analysis for the meta-analysis of cancer-specific survival **(B)**. Begg’s test for the meta-analysis of cancer-specific survival **(C)**.

Seventeen studies with 5,517 patients reported the CSS assessed by multivariate analysis. In view of significant heterogeneity (I^2^ = 64%) in the included studies, a random-effect model was used. The pooled HRs of these studies indicated that the presence of preoperative HN was obviously associated with shorter CSS (HR = 1.71, 95% CI: 1.33 to 2.19, *P* < 0.001) (**Figure 3A**). The sensitivity analysis confirmed the robustness of the results (**Figure 3B**) and the Begg’s test revealed no publication bias among the included studies (*P* = 0.174) (**Figure 3C**).

### Meta-Analysis of DFS

**Figure 4:**
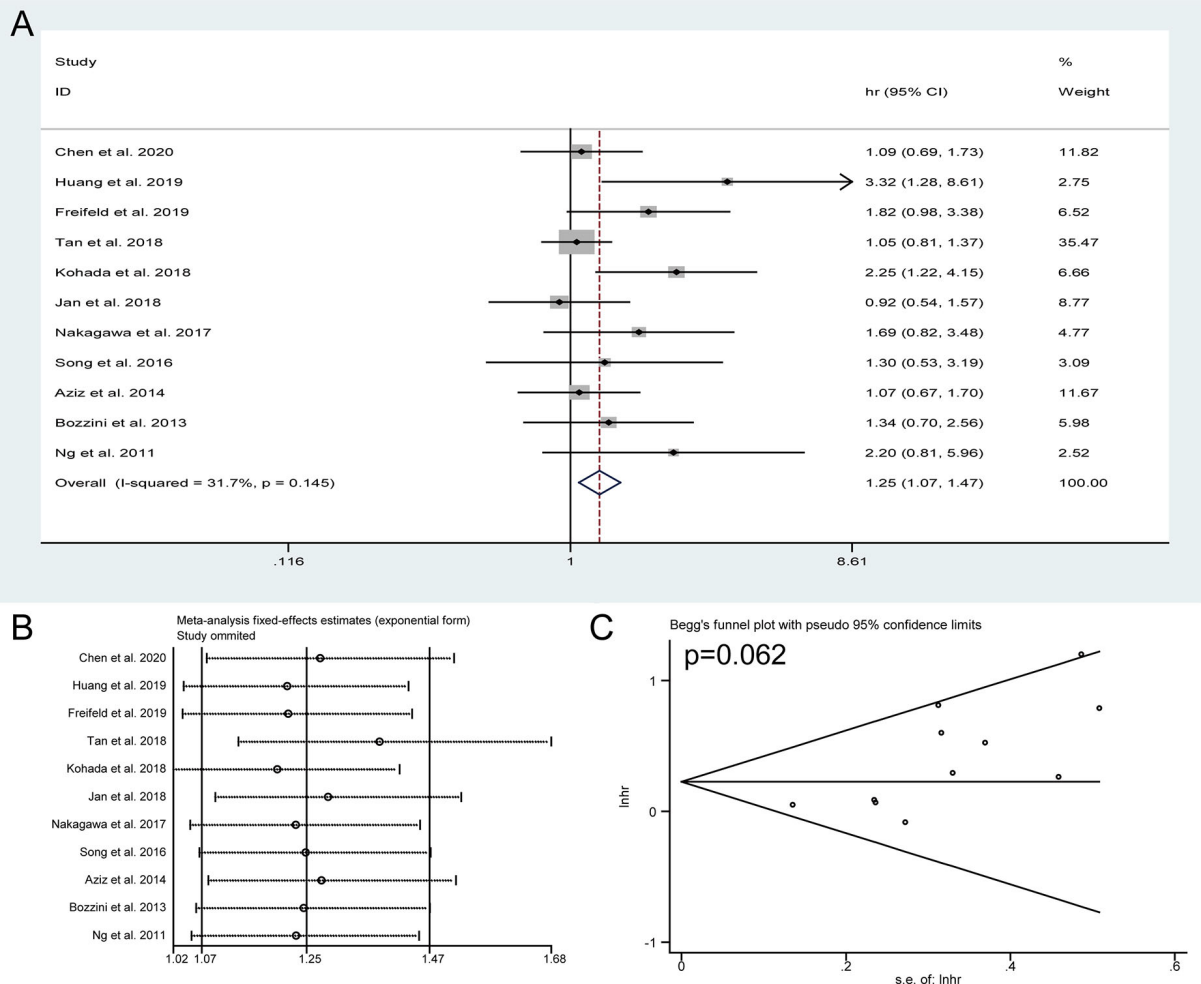
Forest plots of studies to evaluate the relation between preoperative hydronephrosis and disease-free survival **(A)**. Sensitivity analysis for the meta-analysis of disease-free survival **(B)**. Begg’s test for the meta-analysis of disease-free survival **(C)**.

Pooled HRs and 95% CIs of DFS were collected in 11 studies with 2,800 patients. No obvious heterogeneity was detected among studies (I^2^ = 31.7%), and a fixed-effect model was applied to obtain the pooled HRs and corresponding 95% CIs. The result suggested that the presence of preoperative HN predicted a poor outcome of DFS (HR = 1.25, 95% CI: 1.07 to 1.47, *P* = 0.005) (**Figure 4A**). The sensitivity analysis showed that the pooled result of DFS were robust (**Figure 4B**) and Begg’s tests indicated no obvious publication bias in the meta-analysis of DFS (*P* = 0.062) (**Figure 4C**). To discuss the source of significantly worse OS, CSS, and DFS in these patients with preoperative HN, we compared the differences in tumor location, pathologic T stage, lymph node status, and tumor grade between the HN group patients and non-HN group patients, thereby identifying the features between the groups using chi-squared tests for categorical variables (**Table 3**). The results revealed remarkable significant differences in tumor location (*P* < 0.001), pathologic T stage (*P* < 0.001), and lymph node status (*P* = 0.002) between the HN and non-HN groups based on the included studies. However, no obvious difference was found in tumor grade between the two groups (*P* = 0.196)

**Table 3:** Chi-squared tests for the two groups.

Variables	HN group	Non-HN group	*P* value	Study
Tumor location			<0.001	(10, 11, 21, 22, 28, 34),
Pelvis	280	715		
Ureteral	436	197		
pT Stage			<0.001	(10, 11, 21, 34),
≤T2	138	392		
>T2	153	189		
pN Stage			0.002	(10, 11, 21),
pN0/x	113	365		
pN+	38	61		
Tumor grade			0.196	(10, 11, 21),
Low grade or ≤G2	78	231		
High grade or >G2	103	243		

### Meta-Analysis of IVRFS

**Figure 5:**
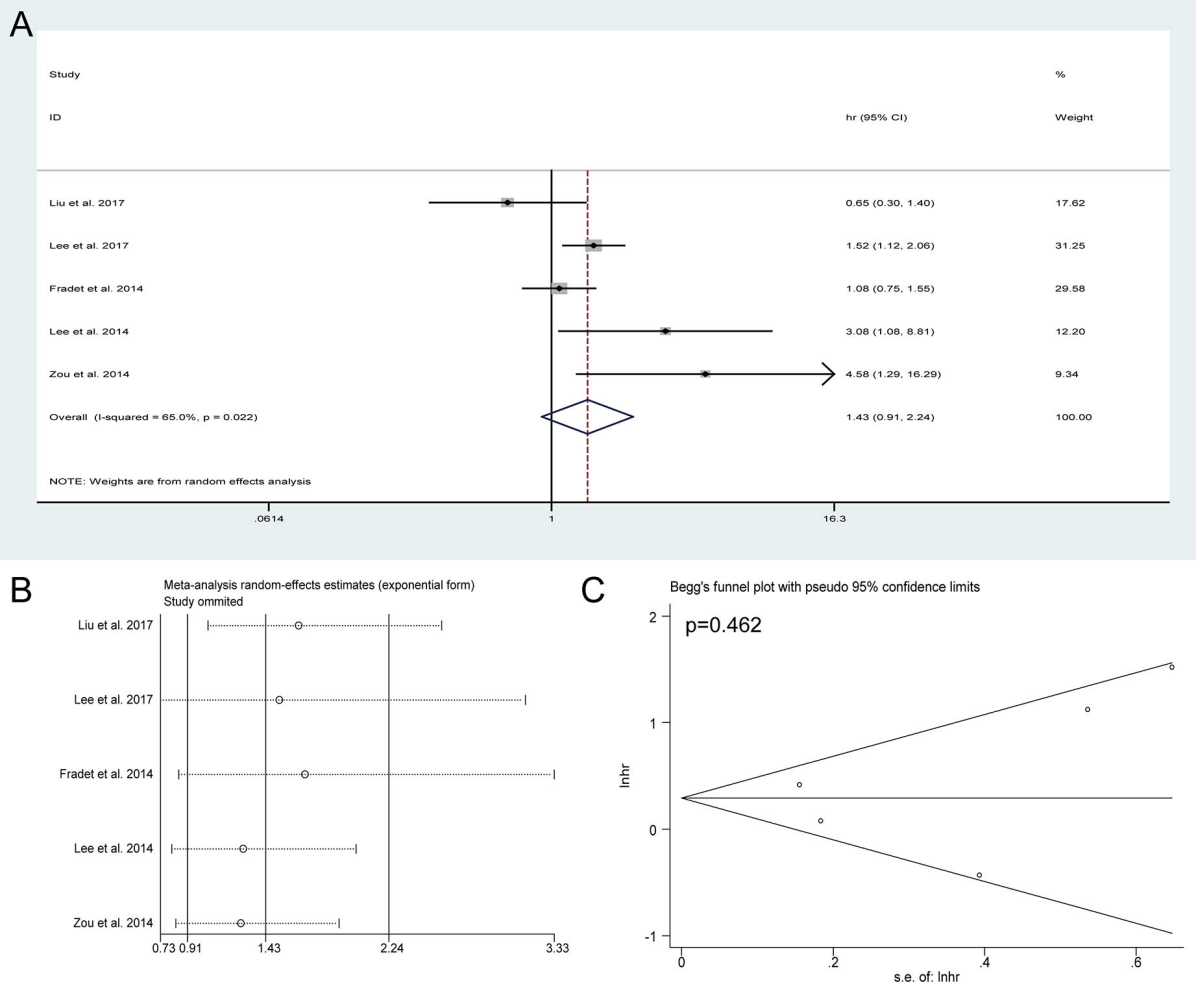
Forest plots of studies to evaluate the relation between preoperative hydronephrosis and intravesical recurrence-free survival **(A)**. Sensitivity analysis for the meta-analysis of intravesical recurrence-free survival **(B)**. Begg’s test for the meta-analysis of intravesical recurrence-free survival **(C)**.

Five studies incorporating 2,027 patients investigated the actual impact of preoperative HN on intravesical recurrence for UTUC after RNU. As present in **Figure 5A**, no significant association was observed between preoperative HN and IVRFS (HR = 1.43, 95% CI: 0.91 to 2.24, *P* = 0.12). The sensitivity analysis showed that the pooled HRs of IVRFS were stable (**Figure 5B**) and Begg’s plot showed no significant evidence of publication bias (**Figure 5C**). These results suggested that preoperative HN could not be used to predict intravesical recurrence for UTUC after RNU based on these included studies.

## Discussion

The association between preoperative HN and oncological outcomes in patients who underwent RNU for UTUC has been widely discussed; however, the results reported by relevant studies were still controversial. Our present meta-analysis of 22 studies including 7,542 patients aimed to investigate the impact of preoperative HN on oncological outcomes for these UTUC patients. The final results revealed the independent predictor status of preoperative HN on OS, CSS, and DFS for patients who underwent RNU for UTUC. However, no significant difference was found when discussing the effect of HN on IVRFS. Besides, we summarized the related data in the eligible studies and found that preoperative HN was significantly associated with ureteral tumors, advanced pT stage, and positive lymph node status, which might contribute to the significant correlation between HN and poor outcomes in UTUC. This correlation will be helpful for guiding decisions about the administration of neoadjuvant chemotherapy.

Recent years, identifying valuable predictors for tumor stage, survival and recurrence of UTUC has attracted extensive interest. Many studies have explored the potential individual or combined implications of various preoperative and postoperative parameters (35). Meta-analysis performed by Wu et al. enrolled 17 articles with 12,094 participants and indicated that ureteral and multifocal tumors could be a prognostic predictor of disease progression and cancer-specific survival for UTUC patients (36). Macroscopic sessile tumor architecture also has been reported to be independently correlated with worse survival after RNU for UTUC (37). Moreover, other valuable prognostic factors also have been identified, and the 2017 EAU guidelines recommended that tumor multifocality, grade on biopsy or cytology, ureteral location, patient age, smoking status, obesity (BMI >30), ECOG-PS ≧1, and delayed surgery (more than 3 months) were valuable preoperative prognosticators (38). However, it remains not very clear for the effect of these potential prognosticators on UTUC patients’ survival. A better understanding of these prognostic factors for tumor progression and patients’ survival would allow more accurate prognostic assessment and more effective therapy approach.

The preoperative pathologic tumor stage, grade, lymph node (LN), and distant metastasis are used as the most crucial prognostic factors for most kinds of malignancies, including UTUC. Despite great advances in medical imaging techniques, its ability to predict preoperative staging and degree of local invasion still has limited accuracy (39), while the preoperative HN status is easily diagnosed for UTUC patients by multiple upper-tract imaging methods, such as CT, MRI, intravenous pyelography, and renal ultrasonography. Several studies have recommended that HN could serve as a potential indicator for invasive disease (>pT2) (40, 41) or non-organ-confined (NOC) disease (pT3–4) (42). Furthermore, it has been identified as a valuable predictor for advanced stage and poor prognosis in transitional cell carcinoma of bladder (43, 44).

Cho et al. reported that the grade of HN on preoperative imaging was a valuable predictor for advanced pathologic tumor stage and worse CSS for ureteral carcinoma (41). Furthermore, both Ng et al. and Zhang et al. similarly found that ureter tumors were more likely to develop HN compared with diseases in renal pelvis, and both the ureter tumor and renal pelvic tumor patients with preoperative HN were correlated with higher tumor stage than patients without HN (11, 34). In addition, several studies revealed that tumor location could be used as an independent prognostic factor of CSS for UTUC patients, and ureter tumors showed worse oncologic outcomes than renal pelvic tumors (34, 45). However, some different voice was also noted. Bozzini et al. conducted a multi-institutional study in France on 401 patients with non-metastatic UTUC and observed no difference in 5 year CSS between HN group (80.1%) and no HN group (83.6%) (*P* > 0.05); moreover, a trend of association between HN and pN stage, not pT stage, was found (*P* = 0.052) (10). Using logistic regression analysis, Liang et al. found preoperative HN had a significant relationship with decreased renal function and LN not LVI; importantly, the Cox analysis results also revealed that HN could serve as an independent risk factor for OS and CSS (28). Otherwise, study by Chung et al. also indicated that HN could predict worse survival for patients with high grade UTUC, not those with low grade UTUC (46).

Overall, preoperative HN can be detected in 37–55% of UTUC patients, while the present knowledge regarding the effect of HN on UTUC prognosis is still inadequate. Our study revealed a significant association of preoperative HN with ureteral tumors, bigger tumor’s size, and positive lymph node status, which might affect the UTUC patients’ prognosis. In addition, it was speculated that HN might cause the outward expansion of high-pressure renal pelvic or ureter wall and increase outward centrifugal pressure, resulting in counter flow in lymphatics and vasculature, which might promote the cancer cells’ seeding to nearby or distant organs (46). To identify the prognostic value of preoperative HN, we performed this meta-analysis and enrolled 22 eligible studies to discuss the pooled effects of HN on oncologic outcomes of UTUC patients after RNU. Preoperative evaluation of advanced stage disease on the basis of HN status will allow urologists make more effective pre- or postoperative treatment strategies. A study about a phase 3, open-label, randomized controlled trial reported that gemcitabine–platinum combination chemotherapy could significantly improve disease-free survival in patients with locally advanced UTUC after nephroureterectomy (47). Earlier use of valid adjuvant therapy will contribute to the improvement in the prognosis for locally advanced UTUC.

Inevitably, our meta-analysis suffered from several limitations. First, all the enrolled studies were retrospectively designed. In spite of the fact that we reached the results by pooling the HRs in multivariate models, some underlying selection bias still existed, and we could not control all the confounding factors. Second, the classification and imaging evaluation method of HN were different: some studies classified HN as “none *versus* present”, but others as “none or mild *versus* severe”; in addition, the definite grade of HN and the influence of different HN grades on prognosis of UTUC patients were not reported in these included studies, which might limit the clinical application of preoperative HN as a adjunctive modality to ureteroscopic biopsy and urinary cytology when counseling UTUC patients on medical and surgical therapies. Third, due to the limited number of included studies, this meta-analysis failed to confirm the value of preoperative HN as a predictor of intravesical recurrence for UTUC after RNU. Finally, this study was performed using the pooled data reported by the eligible studies but not individual patient data, so it is not an individual patient-level meta-analysis. Despite these limitations, our study was the first meta-analysis to identify HN as a potential preoperative prognosticator for UTUC patients undergoing RNU. Well-designed prospective studies are still warranted to further substantiate the prognostic value of preoperative HN in patients with UTUC.

## Conclusions

In this meta-analysis, our results showed that preoperative HN was significantly correlated with worse prognosis in patients with UTUC after RNU, and preoperative HN might serve as an independent prognostic factor. The determination of preoperative HN status might be helpful to identify UTUC patients at higher risk of worse oncological outcomes who could benefit from more aggressive preoperative planning and postoperative therapy.

## Data Availability Statement

The original contributions presented in the study are included in the article/supplementary material. Further inquiries can be directed to the corresponding author.

## Author Contributions

TY and XY analyzed the data and wrote the manuscript. PL and HL searched and collected the literatures. TY and HL contributed to statistical analysis. TY and ZY designed the study and revised the manuscript. All authors contributed to the article and approved the submitted version.

## Conflict of Interest

The authors declare that the research was conducted in the absence of any commercial or financial relationships that could be construed as a potential conflict of interest.
